# Intergenic DNA sequences from the human X chromosome reveal high rates of global gene flow

**DOI:** 10.1186/1471-2156-9-76

**Published:** 2008-11-27

**Authors:** Murray P Cox, August E Woerner, Jeffrey D Wall, Michael F Hammer

**Affiliations:** 1 ARL Division of Biotechnology, University of Arizona, AZ 85721, USA; 2 Institute for Human Genetics, University of California San Francisco, San Francisco, CA 94143, USA

## Abstract

**Background:**

Despite intensive efforts devoted to collecting human polymorphism data, little is known about the role of gene flow in the ancestry of human populations. This is partly because most analyses have applied one of two simple models of population structure, the island model or the splitting model, which make unrealistic biological assumptions.

**Results:**

Here, we analyze 98-kb of DNA sequence from 20 independently evolving intergenic regions on the X chromosome in a sample of 90 humans from six globally diverse populations. We employ an isolation-with-migration (IM) model, which assumes that populations split and subsequently exchange migrants, to independently estimate effective population sizes and migration rates. While the maximum effective size of modern humans is estimated at ~10,000, individual populations vary substantially in size, with African populations tending to be larger (2,300–9,000) than non-African populations (300–3,300). We estimate mean rates of bidirectional gene flow at 4.8 × 10^-4^/generation. Bidirectional migration rates are ~5-fold higher among non-African populations (1.5 × 10^-3^) than among African populations (2.7 × 10^-4^). Interestingly, because effective sizes and migration rates are inversely related in African and non-African populations, population migration rates are similar within Africa and Eurasia (e.g., global mean Nm = 2.4).

**Conclusion:**

We conclude that gene flow has played an important role in structuring global human populations and that migration rates should be incorporated as critical parameters in models of human demography.

## Background

**Figure 1:**
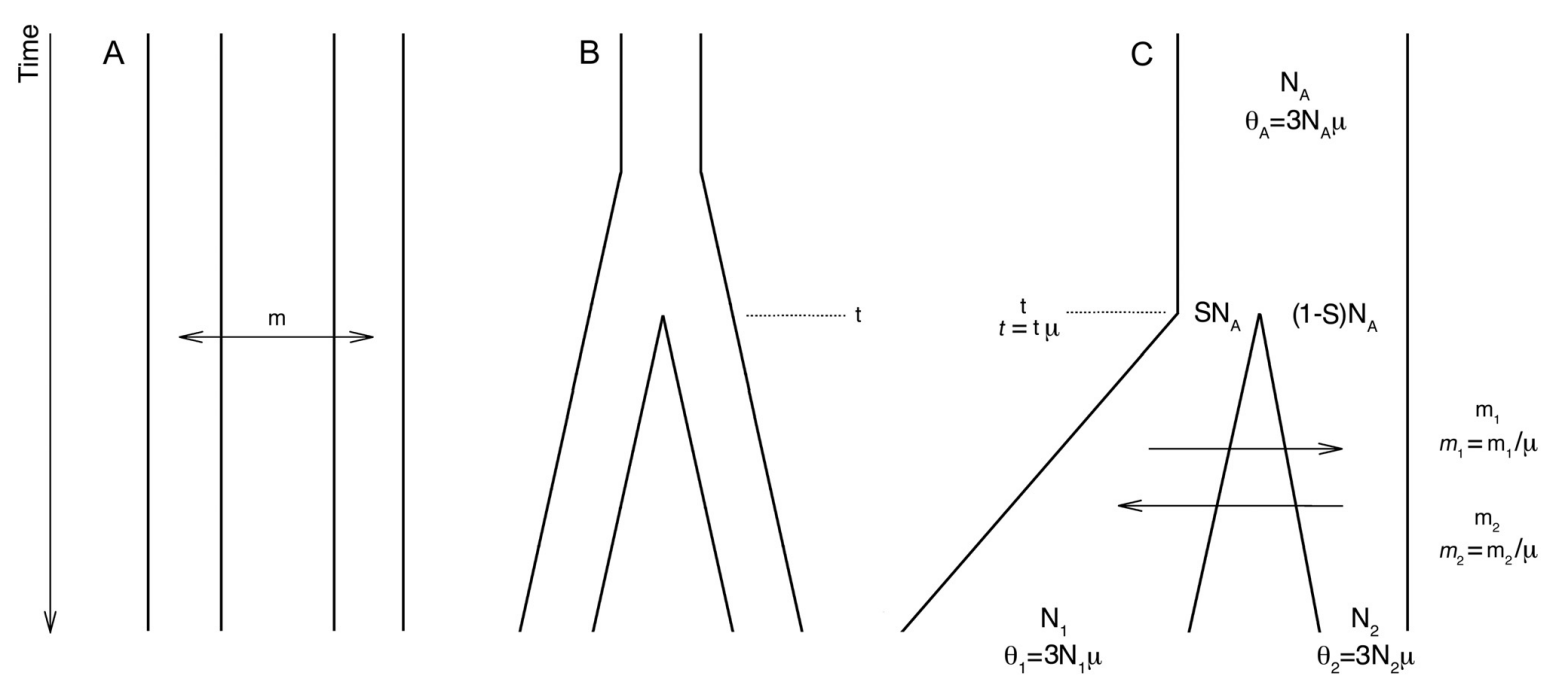
**Models of population structure reflecting the (A) island, (B) splitting and (C) isolation-with-migration (IM) models**. The island model assumes equilibrium gene flow (m) between subpopulations that have no shared ancestry. The divergence model describes an ancestral population, which splits at time t into two daughter populations that do not exchange genes in subsequent generations. The isolation-with-migration model describes a constant-sized ancestral population that splits into two daughter populations that can exchange genes and change in size. There are seven parameters in the isolation-with-migration model: effective population size of the ancestral deme (N_A_), effective population sizes of the two descendent demes (N_1 _and N_2_), unidirectional migration between the descendent populations (m_1 _and m_2_), proportion of the ancestral population founding deme 1 (S), and population divergence time (t).

Reconstructing human history requires an accurate picture of global human population structure [1]. However, methods currently used to describe structure among human groups typically rely on very simple demographic models that make unrealistic biological assumptions. Two commonly used models include the island model, which assumes that populations have no shared ancestry and are related *only *through gene flow (Figure 1A), and the phylogenetic branching or splitting model, which assumes that populations diverged at some time in the past and have remained completely isolated ever since (i.e., no gene flow) (Figure 1B). Despite increasingly sophisticated genetic datasets, most contemporary studies still assume these unrealistic models to infer aspects of human demographic history [2-8].

Furthermore, human population structure is often considered only from the perspective of a single summary statistic, F_ST_, which is a standardized measure of the genetic variation shared between populations. However, F_ST _is dependent on the effective size (N), migration rate (m) and divergence time (t) of individual demes, and by itself has no straightforward demographic interpretation. A small F_ST _between two populations may indicate large effective population sizes, high rates of gene flow since diverging from a common ancestral population, or recent population divergence. For instance, under Wright's [9] island model, N, m and F_ST _are linked by a nonlinear relationship independent of t (equation 2) [10]. Assuming a global effective size for modern humans of ~10^4^, an X chromosome F_ST _of 0.2 [11,12] suggests that human populations have exchanged ~4 gene copies per generation. Conversely, under a simple divergence and isolation model, N, t and F_ST _are related by a nonlinear relationship independent of m (equation 4) [13]. Assuming a mean generation time of 25 years, an X chromosome F_ST _of 0.2 suggests that global human populations diverged on average ~84 Kya. However, despite being in common usage, gene flow-only and divergence-only models probably have little relevance to actual human demographic history.

Here, we examine the structure of human populations by means of the isolation-with-migration (IM) model [14], which incorporates both population splitting *and *gene flow (Figure 1C). The IM model describes two daughter populations, which can expand or contract in size, that diverge from a constant-sized ancestral population with continuing migration between the two demes. The IM model has seven demographic parameters: effective population size of the ancestral deme (N_A_), effective population sizes of the two descendent demes (N_1 _and N_2_), unidirectional migration rates between the descendent populations (m_1 _and m_2_), proportion of the ancestral population founding the first deme (S), and population divergence time (t). Unlike the island model, which assumes infinite divergence times, or the splitting model, which assumes zero migration, IM makes no *a priori *assumptions about these two demographic parameters. It is therefore a more flexible model system for inferring human history from genetic data.

Here, we apply a Bayesian inference framework together with a maximum likelihood algorithm [15] to determine demographic parameters for a range of human populations under the IM model. Marginal Bayesian posterior probabilities are calculated using Markov chain Monte Carlo, and best-fit parameterizations are inferred for each population pair (N = 15) for each of the seven demographic parameters. We analyze a multilocus resequencing dataset comprising 20 intergenic regions on the X chromosome, each of which represents ~5 kb of DNA [16]. To minimize any potential confounding effects of natural selection, the sequenced loci were chosen from single-copy non-coding (i.e., putatively non-functional) DNA in regions of medium or high recombination (r ≥ 0.9 cM/Mb). Such regions are recombinationally unlinked from genic regions. Because we use resequence data (i.e., all sites were sequenced in each individual) and not pre-ascertained single nucleotide polymorphisms (SNPs) [3,17,18], we avoid some biases in aspects of the data that rely on the site frequency spectrum (e.g., F_ST_) [19,20]. These data are also well suited for testing the role of gene flow in the history of anatomically modern humans because multiple, diverse populations are represented in our survey (i.e., Biaka from Central African Republic, Mandenka from Senegal, San from Namibia, Basque from southern France, Han from northern China, and Melanesians from Papua New Guinea).

## Results

### Population differentiation

Wright's F_ST _for our global sample averages to 0.25. When we calculate F_ST _between all pairs of populations, we find the greatest F_ST _values between sub-Saharan African and non-African groups (i.e., F_ST _ranges from 0.160–0.450) (Table 1). However, there is also a substantial amount of differentiation within sub-Saharan African (mean F_ST _of 0.137) and within non-African groups (mean F_ST _of 0.174), with F_ST _values as high as 0.226 between Melanesians and Basque [16].

**Table 1:** Mean effective population sizes, migration rates, divergence times and F_ST_.

	Pop 1	Pop 2	N_A_	N_1_	N_2_	m_12_/gen ×10^-4^	m_21_/gen ×10^-4^	Nm	t (kya)	F_ST_
African										
	BIA	MAN	9,980	3,980	6,600	2.8	1.9	4.9	48.7	0.117
	BIA	SAN	6,620	5,560	5,340	0.86	1.9	3.0	50.0	0.169
	MAN	SAN	9,530	6,930	3,790	0.72	0.020	0.8	46.8	0.126
African/Non-African										
	BIA	BAS	11,200	4,650	3,250	1.1	0.43	1.2	61.4	0.311
	BIA	HAN	12,800	2,330	2,600	0.30	0.075	0.2	27.7	0.374
	BIA	MEL	11,600	6,900	1,570	0.48	0.71	1.0	88.5	0.331
	MAN	BAS	9,970	4,530	2,750	5.8	0.036	4.2	23.4	0.160
	MAN	HAN	11,000	2,750	*nd*	2.2	0.18	*nd*	15.5	0.236
	MAN	MEL	9,420	7,110	318	2.1	3.0	3.8	12.4	0.221
	SAN	BAS	10,400	5,820	2,490	0.42	0.0012	0.3	83.0	0.344
	SAN	HAN	10,200	7,270	1,880	0.24	0.46	0.6	151	0.450
	SAN	MEL	10,500	8,990	1,210	<<0.001	1.6	1.6	68.5	0.390
Non-African/Non-African										
	BAS	HAN	11,900	2,230	1,940	9.2	1.4	4.4	85.6	0.085
	BAS	MEL	11,600	2,120	283	0.26	12	3.0	62.5	0.226
	HAN	MEL	11,300	1,770	592	0.18	21	5.0	61.2	0.210
*Mean*			*10,500*	*3,700*		*2.4*		*2.4*	*59.1*	*0.25*

Abbreviations: BIA, Biaka; MAN, Mandenka; SAN, San; BAS, Basque; HAN, Han; MEL, Melanesians; *nd*: not determined.

### Demographic inference under the isolation-with-migration model

Marginal Bayesian posterior probabilities were calculated using Markov chain Monte Carlo, and best-fit parameterizations were inferred for each unique population pair (e.g., see Figure 1 in Additional file 1). Results for the seven demographic parameters are discussed individually below (also see Table 1).

#### Effective population sizes

Modern effective population sizes (N) were inferred multiple times under the IM model (i.e., once for each paired population, Tables 1 and 2 in Additional file 1). Although individual estimates of N have some uncertainty, mean effective sizes are statistically larger for African populations (2,300–9,000) than non-African (300–3,300) populations (*t*_20 _= 6.9, *P *<< 0.001). Some non-African groups have especially small effective sizes, such as Melanesians (N_0 _<1,500). Mean ancestral sizes (10,500, range 6,600–12,800) are generally larger than modern effective sizes, and are also often larger than the sum of their descendant populations (*t*_19 _= 3.42, *P *= 0.0029).

#### Population split proportions

Our dataset has little power to infer how ancestral effective sizes were apportioned among descendent demes. Most estimates of the split proportion, S, have large confidence intervals (Table 3 in Additional file 1). The few informative estimates indicate substantial retention of ancestral effective size in African populations; for instance, less than ~10% of the ancestral population size of Mandenka and Papua New Guineans is retained in the latter population today. Similarly, among non-Africans, the Han Chinese retain less than ~10% of the diversity carried when their ancestral deme still included the ancestors of modern Papua New Guineans (upper bounds of the 95% confidence interval for S).

#### Migration rates

Stationary estimates of long-term unidirectional migration rates average to 2.4 × 10^-4^/generation (range: 8.7 × 10^-8 ^– 2.1 × 10^-3^/generation), thereby suggesting that gene flow between global populations is relatively frequent (Table 4 in Additional file 1). The highest bidirectional migration rate (2.1 × 10^-3^; lowest rate: 3.8 × 10^-5^) implies a movement of ~1 X chromosome every 2 years. Bidirectional migration rates within and between continents vary significantly (*F*_2,12 _= 21.5, *P *= 0.0001, *η *= 0.78), with continental assignment accounting for 61% of the variation in migration rates. Mean bidirectional migration rates within Africa (2.7 × 10^-4^), and between Africans and Eurasians (2.1 × 10^-4^), are relatively low compared to migration rates within Eurasia (1.5 × 10^-3^). Furthermore, migration patterns between populations are largely symmetric. Han Chinese and Melanesians provide a key exception; migration from China to the Pacific (2.1 × 10^-3^/generation) has significantly exceeded migration in the opposite direction (1.8 × 10^-5^/generation) (see 95% confidence intervals in Table 4 in Additional file 1).

#### Population divergence times

Marginal posterior distributions for t indicate that divergence times between African populations all occur ~50 kya, with the largest upper confidence interval for any two African populations at ~140 kya (Table 5 in Additional file 1. Estimates of non-African split times are made difficult by diffuse posterior distributions with very high variance, and thus caution is advised in interpreting the higher mean non-African divergence time (~67 kya). Estimates of the lower bounds for non-African divergence times, with a mean of ~30 kya, are more consistent with previous estimates. The mean divergence time between African and non-African populations is ~58 kya (or ~2,100 generations), which is very similar to previous estimates of the out-of-Africa expansion inferred from other genetic data [21-23].

### Validation of inferred demographic parameters

The inference system employed here has been validated elsewhere [15], and our own tests indicate that known demographic parameters (including m) for data simulated under an IM model are recovered within 95% confidence intervals (unpublished data). However, to further assess the suitability of the IM model to explain observed patterns of variation, we used coalescent simulation to model each population pair (N = 15) using the parameter values of N_A_, N_1_, N_2_, m_1_, m_2 _and t inferred above. Due to poor estimates of the split proportion, we assumed S = 0.5. To check whether these coalescent simulations return data similar to the empirical loci, we compared observed summary statistics for all twenty X chromosome loci with summary distributions from these parameterized simulation models. We focused on four summaries of the data: i) F_ST_, which describes the genetic distance between populations; ii and iii) *θ*_W _and *θ*_*π*_, which are unbiased estimators of the population mutation rate *θ *(= 3N_e_*μ*); and iv) Tajima's D, which summarizes the site frequency spectrum.

**Figure 2:**
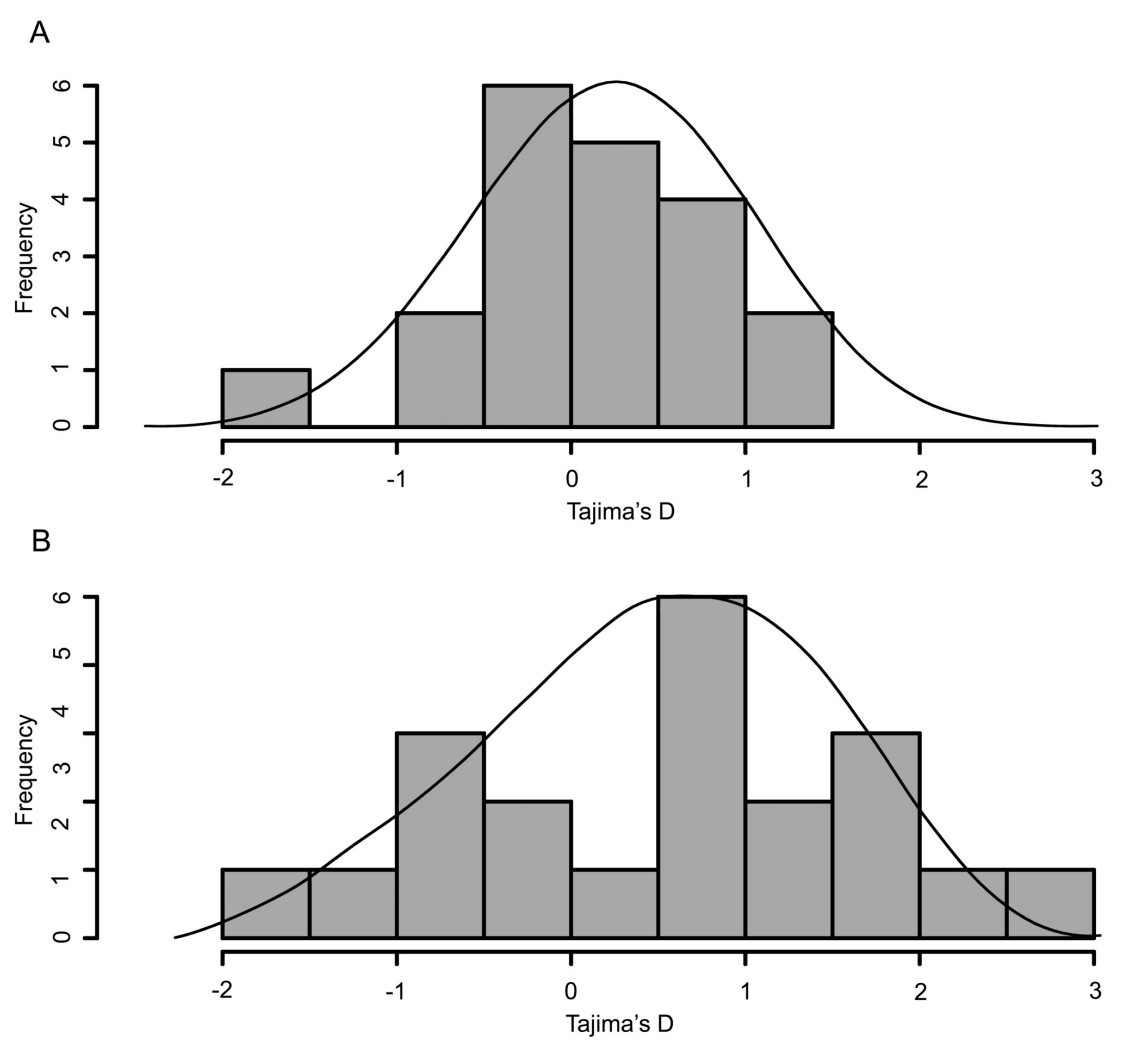
**Observed values (bars) of Tajima's D for 20 X chromosome loci in (A) Biaka pygmies and (B) Melanesians compared to simulated distributions for each population (curves)**. Tajima's D values for the majority of empirical loci are consistent with simulated distributions that are obtained from an isolation-with-migration model parameterized with the inferred demography of these two populations.

Observed F_ST _values are correlated with F_ST _values simulated under these 15 simulation models (Mantel test, r = 0.49, *P *= 0.039). Although this only explains 24% of the variance, we obtain mean F_ST _values that are just slightly lower than those actually observed (i.e., mean F_ST _of 0.21 versus 0.25, not significantly different). The simulation models also provide good fits to observed data for the remaining summaries, all of which reflect aspects of the population site frequency spectrum. A Bonferroni correction holding the experiment-wise type-I error rate constant at *α *= 0.05 was applied to accommodate multiple tests for each population and each summary. We observe 2% of loci as outliers under the corrected 95% confidence intervals for the 360 tests performed on our parameterized simulation models (i.e., 6 populations, 20 loci, and 3 summary statistics) (Table 6 in Additional file 1). This degree of consistency between observed and simulated data is similar for both African and non-African populations (e.g., Figure 2). This similarity suggests that our estimates of demographic parameters are not strongly biased by the inference method, and that the IM model is capable of portraying the evolutionary processes underlying human population structure, at least at the geographic scale examined here [24].

## Discussion

It is generally appreciated that migration affects many important ecological and evolutionary properties of populations [25]. Yet there is little consensus in the literature on the role of gene flow in shaping patterns of human population structure. Equilibrium models, like the island model [9], were favored historically because analytical expectations are relatively simple to obtain. On the other hand, while it is accepted that migration occurs frequently among human populations [26-31], gene flow is often ignored in models of human demography. Recent examples ignoring gene flow include studies of African replacement models [32] and serial-founder models for the settlement of Eurasia [5]. Other studies of human demographic history analyze data separately for each population sample and simply neglect any effects of shared common ancestry or gene flow [7].

As pointed out by Whitlock and McCauley [25], both direct and indirect measures of migration and gene flow are fraught with difficulty. The use of summary statistics such as F_ST _is limiting because it provides no insight into which historical processes are responsible for the observed genetic differences between populations. It is therefore left up to individual investigators to choose which model of population structure is used to interpret data, and inferences depend heavily upon the assumptions inherent in each model. We set out to directly estimate rates of gene flow between human populations through the use of the isolation-with-migration (IM) model, which incorporates both population splitting and gene flow. For this purpose, we have analyzed a large DNA sequence database collected with the expressed purpose of constructing models of human demographic history, and hence, is focused on intergenic (i.e., putatively neutral) regions on the X chromosome [16]. Values of F_ST _for this dataset were found to be slightly higher than those estimated from other large X chromosome resequence datasets [11,12]. This difference is probably driven, at least in part, by the inclusion of more isolated groups than were included in other DNA sequence surveys of X-linked loci (i.e., the San from Namibia, Biaka from Central African Republic, and Melanesians from Papua New Guinea) [16].

To disentangle the evolutionary processes underlying F_ST _in real human populations, we inferred N, m and t separately for the six populations in our survey using the IM model. While the mean global value for ancestral population size, ~10^4^, is consistent with previous estimates of the global population size of modern humans [33-37], no individual population approaches the effective size of modern humans as a whole. Although sizes of individual populations estimated from nuclear loci remain sparse, other studies have produced similar results [2,38-40], including previous applications of the IM model to human genomic sequences [41]. Non-African population sizes (N_0 _≈ 3000) estimated from linkage disequilibrium (LD) [42] are particularly noteworthy because recombination information is independent of the site frequency spectrum, which we used to infer effective sizes here. That similar results are obtained using unrelated subsets of the genetic information, and different populations, reinforces our confidence in these low estimates of effective population size.

This finding is important because many studies make the simplifying assumption that individual human populations have an effective population size of 10^4 ^[e.g., [43]]. We note that estimates of N based on the standard neutral model (i.e., *θ *= 3N*μ*) are 2–4 times higher than those returned by IM for individual African and Eurasian samples, and are even higher for our Melanesian sample (see Table 1 in Wall et al. [16]). We suspect that this difference is largely due to violations of assumptions of the Wright-Fisher model rather than a systematic bias in parameters returned by IM (see Results). For example, population substructure and gene flow are expected to upwardly bias estimates of within population diversity (e.g., *θ*) when applying models that do not incorporate these variables.

Indeed, we demonstrate here that rates of gene flow between subdivided human populations are non-zero. For unidirectional migration rates (m_1_, m_2_), ~87% of pairwise comparisons (i.e., 42 of 48) showed gene flow in at least one direction (i.e., their 95% confidence intervals exclude zero migration; Table 4 in Additional file 1). When bidirectional migration rates (m = m_1 _+ m_2_) are considered, lower bounds of 95% confidence intervals are greater than zero for all 15 pairwise comparisons. Furthermore, mean bidirectional migration rates within Africa (2.7 × 10^-4^/generation), and between Africans and Eurasians (2.1 × 10^-4^), are significantly lower than migration rates within Eurasia (1.5 × 10^-3^).

**Figure 3:**
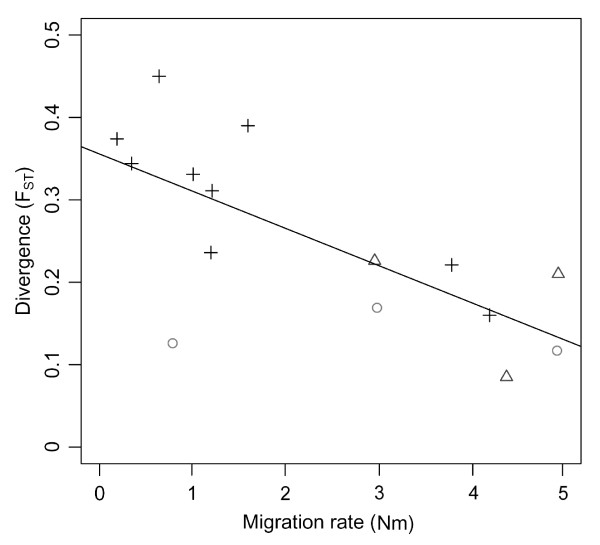
**Correlation between population divergence (F_ST_) and inter-deme migration (Nm)**. African population pairs are indicated by circles, non-African population pairs by triangles, and African/non-African population pairs by crosses.

**Figure 4:**
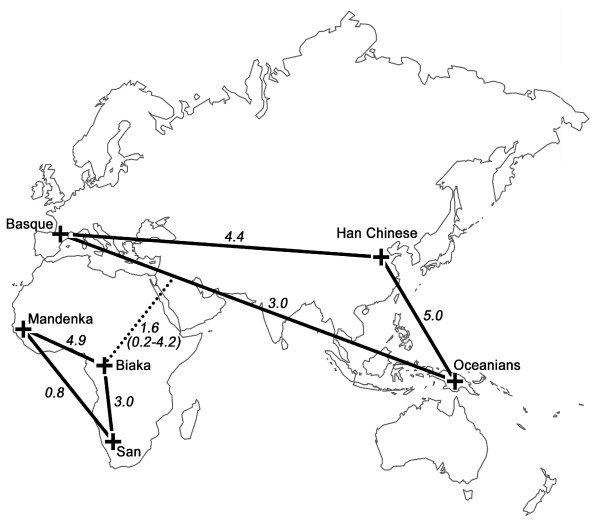
**Geographic representation of population migration rates Nm**. Mean and range of Nm are provided for African/non-African population pairs.

Correspondingly, estimates of the population migration rate range from 0.2–4.9 (mean Nm = 2.4). This implies that 2–3 X chromosome copies, on average, move between human populations every generation, although this stationary estimate does not explain how migration events are distributed through time. We infer slightly higher population migration rates within Africa and within Eurasia than between continents (Figure 3). Interestingly, because effective sizes and migration rates are inversely related in African and non-African populations, values of Nm are similar within Africa and Eurasia (Figure 4). Furthermore, migration rates are not associated with geographic proximity in any simple fashion. For instance, Basque and Han are located far apart geographically, but exhibit one of the highest rates of gene flow. This presumably reflects factors affecting historical mobility that are more complex than simple isolation-by-distance.

Clearly, our results cannot be interpreted under either the pure splitting or island models, which assume no gene flow and no shared ancestry, respectively. In any case, under an island model, a population migration rate Nm greater than ~0.25 would be too high to explain the value of F_ST _observed here (cf. Figure 2A in Additional file 1). Indeed, no further differentiation of local island populations is expected when Nm > 1 [44,45]. Note, however, that the axiom whereby exchanging one gene copy per generation inhibits population divergence does not hold for the IM model; populations can continue to diverge, albeit slowly, even when Nm = 3.3 (Figure 2C in Additional file 1). Importantly too, F_ST _depends on interactions between a suite of demographic parameters, including N, m and t (Figure 3 in Additional file 1). These results highlight the limited power of F_ST _to yield insights into human demographic processes without further knowledge of population divergence times, effective sizes and migration rates (i.e., the very parameters that we often attempt to infer from F_ST_). Although F_ST _is often considered directly proportional to divergence time, where migration is assumed to be absent and all population sizes identical [46:29–30], these assumptions do not hold for the human populations examined here. Thus, caution is warranted when interpreting F_ST _as a simple proxy for population history [e.g., [5,46]].

In sum, we have independently inferred effective population sizes, times of divergence and rates of migration under an IM model of population structure based on the analysis of a large X chromosome DNA sequence database. The parameters that we have estimated for six globally distributed populations indicate relatively high levels of migration (e.g., mean Nm = 2.4) (Figure 4). A simple interpretation of our results is that human populations have exchanged migrants at a constant high rate during the entire history since their divergence from a common ancestral population. A more realistic interpretation is that they have experienced changing migration rates through time. Unfortunately, we cannot currently discriminate between these two scenarios; IM can only infer a stationary rate of gene flow (i.e., a time averaged rate). Our initial explorations permitting migration rates to change through time suggest that this process can have significant effects on the value of F_ST _(e.g., see Figure 4 in Additional file 1). There is also independent evidence that rates of gene flow may be increasing towards the present. For example, the very large and asymmetric gene flow that we observe between Chinese Han and Melanesians may well result from the late Holocene (3–4 kya) expansion of agricultural populations recorded not only by haploid genetic loci [47,48], but also archaeologically and linguistically [49]. This is similar to Wakeley's [50] inference based on a maximum likelihood analysis of restriction fragment length polymorphisms (RFLPs) from across the genome that human populations have transitioned from a period of higher to lower population structure through an increased rate of migration towards the present. We also acknowledge that long-term mean rates of migration may be affected by both recurrent gene flow restricted by geographical distance [51] and long range dispersals by groups of migrants (i.e., range expansions), which can occur sporadically and still have large effects on global values of F_ST _[29,52,53]. Interestingly, population genetic models with spatial and temporal fluctuation in the population migration rate (averaging around Nm ~1 in an island model) have been shown to be conducive both to the rapid spread of beneficial mutations throughout the species and local population differentiation [54]. We note that theoretical predictions based upon other models of subdivision (such as metapopulation models) [8,52] would be worth developing to further explore the nature and extent of human population structure.

Finally, the finding of high rates of gene flow among human populations has important implications for how we interpret the distribution of SNPs associated with disease [55] and the role of natural selection in shaping patterns of diversity [31,52,53]. For instance, recent migration produces haplotypes with extended linkage disequilibrium that could be misinterpreted as recent selection [56]. Gene flow can also significantly skew the site frequency spectrum within populations [8], and may therefore lead to erroneous inferences regarding demography.

## Conclusion

While the maximum effective size of modern humans is estimated at ~10,000, individual populations vary substantially in size. African populations tend to be larger (2,300–9,000) than non-African populations (300–3,300). We independently estimate mean rates of bidirectional gene flow at 4.8 × 10^-4^/generation, and these rates are higher among non-African populations (1.5 × 10^-3^) than among African populations (2.7 × 10^-4^). Interestingly, because effective sizes and migration rates are inversely related in African and non-African populations, effective migration rates are similar globally (e.g., mean Nm = 2.4). While significant theoretical challenges remain in disentangling the evolutionary factors that structure human populations, it is clear that migration can no longer be treated as a simple, equilibrium parameter – or ignored – as it often is in reconstructions of human history.

## Methods

### Genomic data

Our database comprises 20 loci from intergenic regions on the X chromosome. Each region chosen for sequencing spans ~20 kb of primarily single-copy non-coding (i.e., putatively non-functional) DNA in regions of medium or high recombination, which are at least 50 kb away and recombinationally unlinked from the nearest gene [16]. No two regions are within one megabase of each other. Within each region, there are ~4–6 kb of sequence data from 3 discrete subsections that span most of the distance of each region. 10–24 X chromosomes are sampled from each population, which include 3 from Africa (10 San from Namibia, 16 Biaka pygmies from Central African Republic, 18 Mandenka from Senegal) and 3 from Eurasia/Oceania (16 French Basque, 16 Han Chinese, 24 Melanesians) (see Wall et al. [16] for a complete description of the sequencing strategy).

### F_ST _estimates

F_ST _can be calculated using several different algorithms. Here, we adopt the approach of Hudson et al. [57], defined in terms of polymorphic site heterozygosity, which we have amended to accommodate unequal sample sizes [58] and missing data.

(1) $${\text{F}}_{\text{ST}}=\text{1}-\frac{{\text{H}}_{\text{w}}}{{\text{H}}_{\text{b}}}=\text{1}-\frac{\pi_{\text{w}}}{\pi_{\text{b}}}$$

where H_w _(≡ *π*_w_) is the mean distance per polymorphic site sampled from the same population, and H_b _(≡ *π*_b_) is the mean distance per polymorphic site sampled from both populations. Reported values represent the mean F_ST _at all segregating sites across all 20 X chromosome loci.

The expected value of F_ST _for X chromosome loci under the island model with an infinite number of demes depends only on the product of the effective population size N, and the migration rate per generation m [10: 294-5]

(2) ⟨F_ST_⟩ ≈ (1 + 3 Nm)^-1^

F_ST _estimates must be corrected if a finite number of demes d are intended instead [59]

(3) $$\langle {\text{F}}_{\text{ST}}\rangle \approx {\left(\text{1}+\frac{{\text{d}}^{\text{2}}}{{\left(\text{d}-\text{1}\right)}^{\text{2}}}\left(\text{3Nm}\right)\right)}^{-1}$$

The population-scaled rate of gene flow Nm can be derived by simple rearrangement of equation 3.

Correspondingly, the expected value of F_ST _for X chromosome loci under a divergence model depends only on the divergence time t, in generations, scaled by the effective population size N [13]

(4) 〈FST〉=1−e−t32N

### Demographic inference

Genetic diversity at twenty X chromosome loci is applied to determine the most likely parameterization for a series of paired population isolation-with-migration models. Seven demographic parameters are inferred from the genomic data under each two-deme IM model: effective population size of the ancestral deme (N_A_), effective population sizes of the two descendent demes (N_1 _and N_2_), unidirectional migration rates between descendent populations (m_1 _and m_2_), proportion of the ancestral population founding the first deme (S), and population divergence time (t). Populations are analyzed in all pairwise combinations using the Markov chain Monte Carlo Bayesian/maximum likelihood framework implemented in the 31 July 2006 version of IM . More complete descriptions of this method are available elsewhere [14,15,41,60,61]. The IM software infers all coalescent parameters, except S, as mutation-scaled rates – *θ*_1 _= 3N_1_*μ*, *θ*_2 _= 3N_2_*μ*, *θ*_A _= 3N_A_*μ*, *m*_1 _= m_1_/*μ*, *m*_2 _= m_2_/*μ*, and *t *= t*μ *– which, if mutation rates are known, can subsequently be transformed to real world values (i.e., actual population sizes, migration rates and chronological dates). Mutation rates per year are estimated for each locus from mean *Homo*/*Pan *sequence divergence assuming a split time of 6 × 10^6 ^years. Per generation rates assume a mean generation interval of 28 years, as estimated from cross-cultural ethnographic data [62]. A 1:1 mating ratio is also assumed throughout. Migration rates m_1 _and m_2 _are inferred in the coalescent (i.e., backward in time), and bidirectional migration rates m are simply the summation of m_1 _and m_2_.

Because the IM algorithm has most power with perfectly treelike data (an infinite sites implementation), datasets with no evidence of recombination were extracted from each locus using the four-gamete approach of Hudson and Kaplan [63]. Individuals and segregating sites are given equal weighting, and the largest non-recombining block maximizing overall information content is selected for each locus, a practice that minimizes any bias in the resulting dataset [64]. Analyses are run at a mean CPU speed of 2.5 GHz on an ~100 core Condor grid at the University of Arizona Computer Science Department. All datasets are initially parameterized from a single run with bounded uniform priors: *θ*_1_, *θ*_2_, *θ*_A _∈ *U*(0, 40), *m*_1_, *m*_2 _∈ *U*(0, 20), *S *∈ *U*(0, 1) and *t *∈ *U*(0, 3). Ranges are raised or lowered in subsequent runs to incorporate complete marginal posterior probability densities. Once these bounds are established, a minimum of four replicate jobs each of >5 chains are run for a minimum of 10^7 ^steps. Chain mixing by Metropolis-Hasting coupling, long run times and multiple independent runs allow us to identify convergence on each parameter's underlying stationary distribution.

Because we observe little variation among multiple independent runs (e.g., Figure 1 in Additional file 1), marginal posterior probability densities for a given parameter are combined and analyzed as a single probability distribution. Maximum probability estimates and 95% confidence intervals are reported as modes and {0.025, 0.975} quantiles of posterior probability densities, respectively. We estimate all seven parameters of the model (Tables 1–5 in Additional file 1) for all population pairs. Uninformative posterior distributions are sometimes encountered for some population pairs; in particular, the ancestral population split time parameter t often proves difficult to infer (Table 5 in Additional file 1). However, the seven demographic parameters are inferred as marginal densities, and therefore uncertainty in one parameter has no impact on the remaining parameters. Parameter values are treated as unknown unless we observe clear maximum likelihood peaks that are replicated across multiple independent IM jobs.

### Coalescent simulations for demographic parameter validation

The nonlinear relationship between gene flow, divergence time and F_ST _under the isolation-with-migration model is explored using coalescent simulation with the software ms [65]. An IM model conditioned on mean values for the seven demographic parameters, as inferred from the empirical dataset (Tables 1–5 in Additional file 1), is implemented for each population pair. A suite of summary statistics is explored under these models: *θ*_W _and *θ*_*π*_, which are unbiased estimators of the population mutation rate; Tajima's D, which summarizes the population site frequency spectrum; and F_ST_, which summarizes the joint site frequency spectrum. Observed values of these four summary statistics, calculated from the empirical dataset, are compared to the summary statistic distributions returned by coalescent simulation. A Bonferroni correction holding the experiment-wise type-I error rate constant at *α *= 0.05 is applied to account for the use of multiple per-population tests. A Mantel test is used to determine the correlation for F_ST_, a between-population (i.e., matrix) test.

## Authors' contributions

MPC participated in the design of the study, contributed to data collection, ran analyses, and wrote the manuscript. AEW contributed to data collection. JDW advised on data analysis, and provided comments on the manuscript. MFH participated in the design of the study, advised on data analysis, and helped revise the manuscript. All authors read and approved the final manuscript.

## Supplementary Material

Additional file 1 **Supplemental Materials for "Intergenic DNA sequences from the human X chromosome reveal high rates of global gene flow".** This document contains tables and figures showing additional results referenced in the main text. Click here for file
