# Supplementary material for: Intergenic DNA sequences from the human X chromosome reveal high rates of global gene flow
Source: BMC Genet. 2008 Nov 27;9:76. doi: 10.1186/1471-2156-9-76 (PMC2620354; doi:10.1186/1471-2156-9-76)
Supplement: Additional file 1 — Supplemental Materials for "Intergenic DNA sequences from the human X chromosome reveal high rates of global gene flow". This document contains tables and figures showing additional results referenced in the main text. [file 1471-2156-9-76-S1.pdf]

Supplemental Materials for:

Intergenic DNA sequences from the human X chromosome reveal high rates of global gene flow

Murray P Cox, August E Woerner, Jeffrey D Wall and Michael F Hammer

Supplemental Figure Legends

Supplemental Figure 1. Example IM results. Marginal posterior densities of demographic parameters inferred for the Mandenka-San population pair.

Supplemental Figure 2. Population divergence versus constant gene flow for the (A) island, (B) splitting, and (C) isolation-with-migration (IM) models. Dotted, solid and dashed lines reflect constant population migration rates (Nm) of 0.033, 0.33 and 3.3, respectively. Constant sized populations of $N_0 = N_1 = 3{,}000$ were simulated. Populations diverged 2400 generations ago, or ~60 kya. F_{ST} for the island model was calculated with equation 3 (where d = 2), F_{ST} for the splitting model was calculated with equation 4, and F_{ST} for the isolation-with-migration model was estimated by coalescent simulation. Note that the divergence model is a special case of the isolation-with-migration model such that Nm = 0. The F_{ST} curves in pane C can be viewed as resulting from different effective sizes (N), or different migration rates (m), or a combination of both parameters (i.e., Nm).

Supplemental Figure 3. Interaction between rates of gene flow and population divergence times relative to F_{ST} for X chromosome loci. (A) Parameter bounds determined by IM for real human populations fall within the large box; contour lines demark parameter space producing specific F_{ST} values (e.g., $F_{ST} = 0.1$). F_{ST} surfaces were generated by coalescent simulation under an isolation-with-migration model with constant-sized demes. (B) F_{ST} surface detail. The leftmost box shows the parameter space where combinations of gene flow and divergence most

closely approximate a divergence model (for F_{ST} = 0.1); the rightmost box shows where combinations of gene flow and divergence most closely approximate an island model (also for F_{ST} = 0.1). The intervening curve is a poor fit to either model.

Supplemental Figure 4. Population divergence, F_{ST}, versus changing rates of gene flow, Nm, through time. Graphs illustrate the effects of migration rates (A) decreasing exponentially, (B) increasing exponentially, (C) increasing instantaneously 10 kya in the Holocene, and (D) increasing instantaneously 30 kya in the Pleistocene. Dotted, solid and dashed lines reflect changing migration rates (Nm) with long-term averages of 0.033, 0.33 and 3.3, respectively. The total quantity of migration does not differ between graph lines representing the same value of Nm.

Supplemental Figure 1

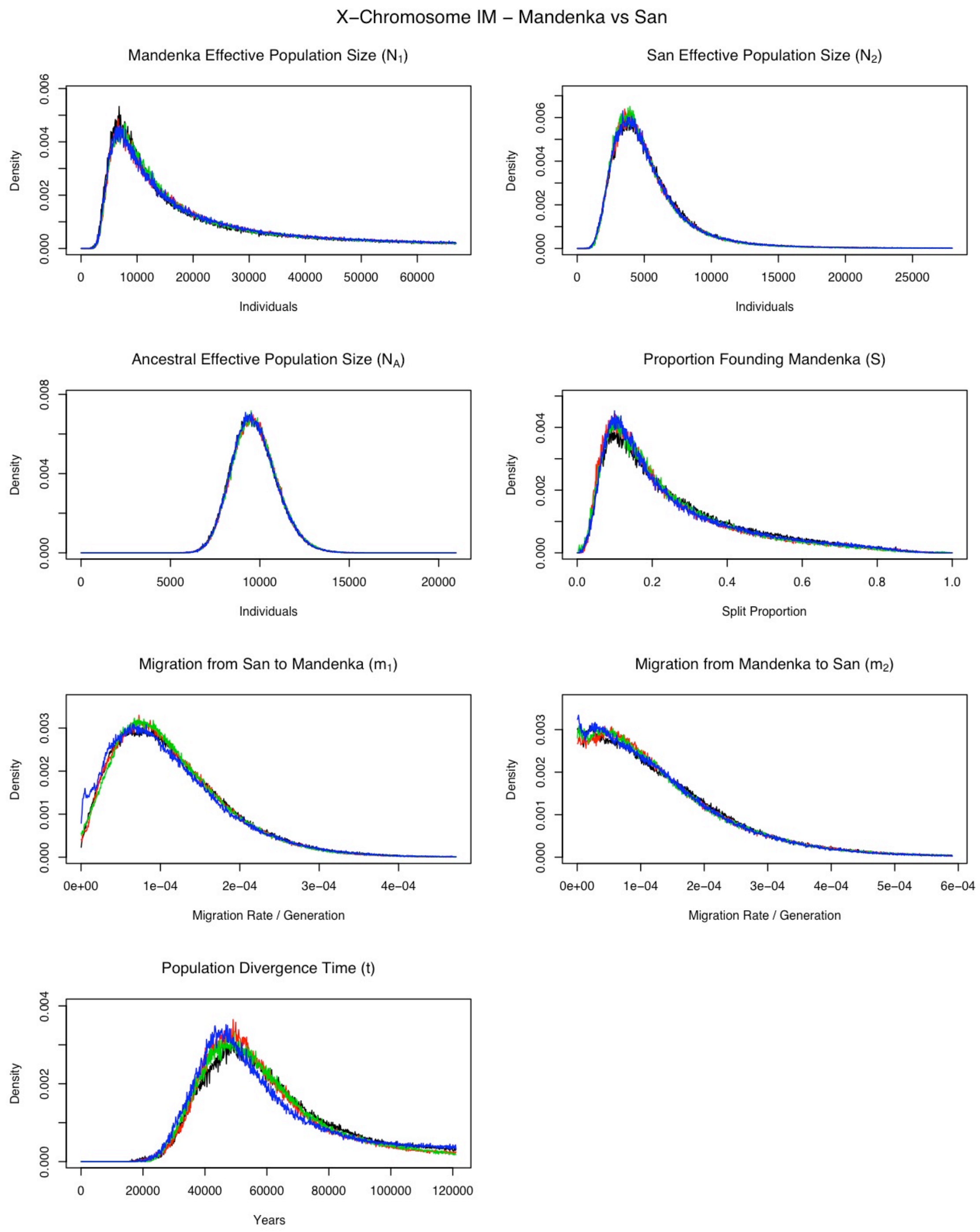

Supplemental Figure 2

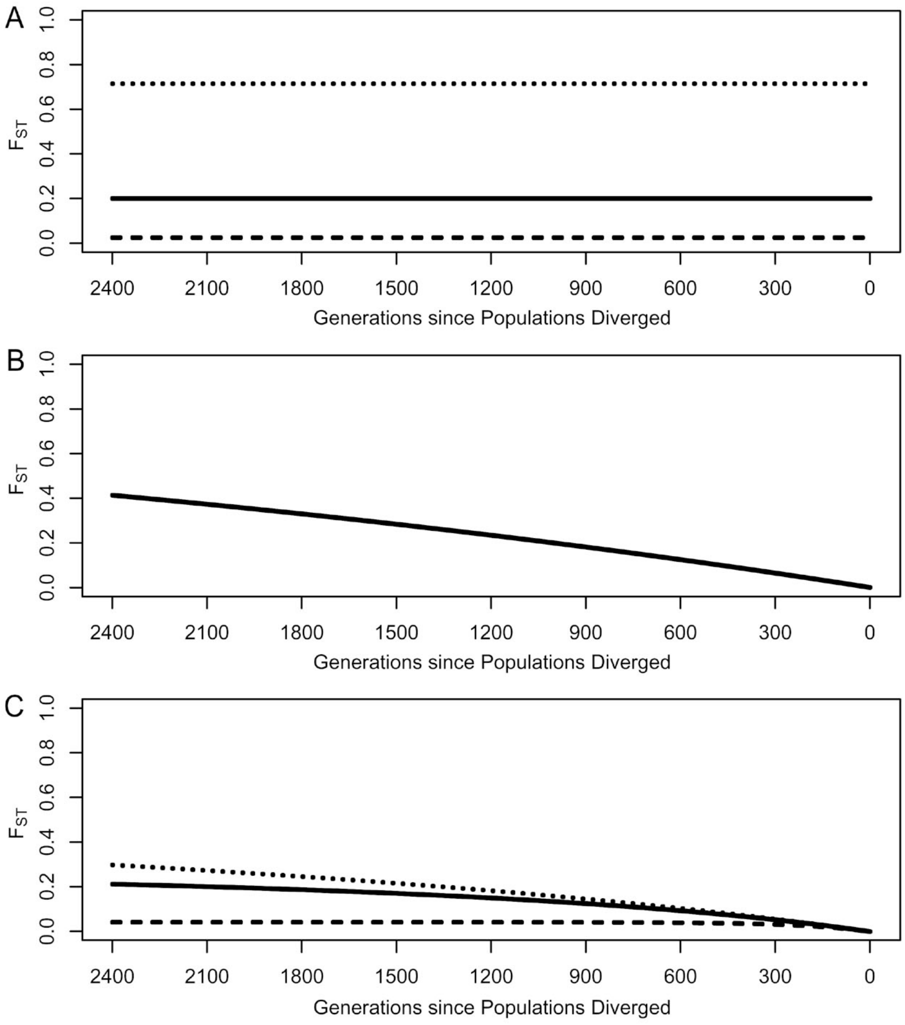

Supplemental Figure 3

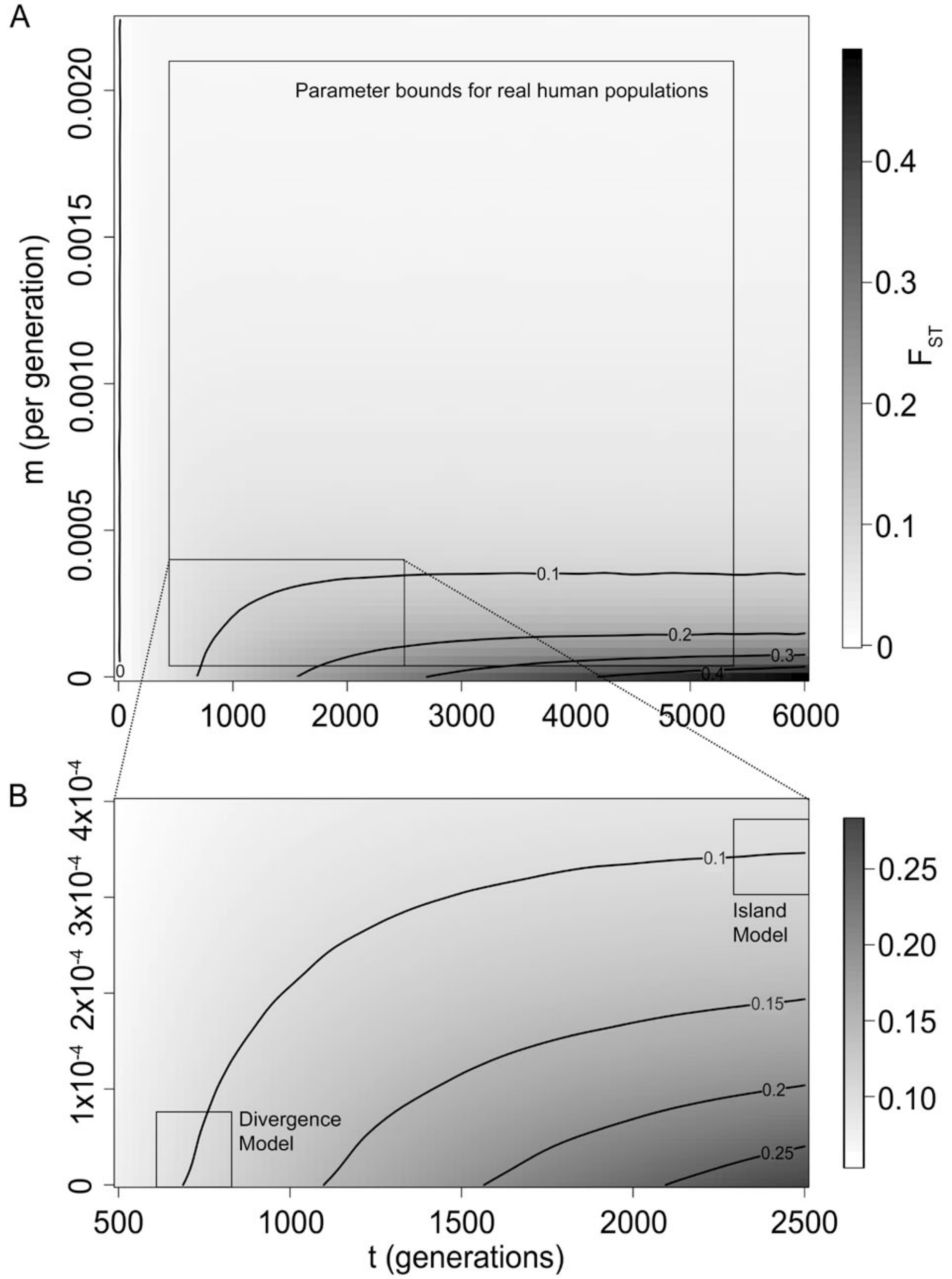

Supplemental Figure 4

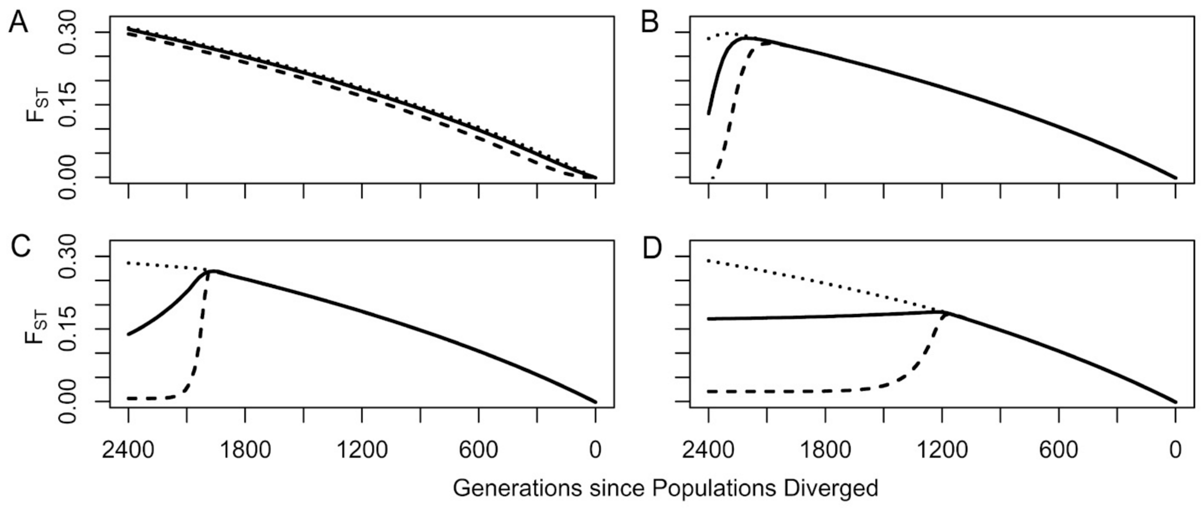

Supplemental Table 1. Modern effective population sizes (N). Multiple pairwise estimates of N are listed for each population on the horizontal. Central 95% confidence limits in parentheses. Abbreviations: *nd*, not determined.

	**Biaka**	**Mandenka**	**San**	**Basque**	**Han Chinese**	**Melanesians**	**PNG**
**Biaka**	—	3980 (2060–7820)	5560 (3160–9940)	4650 (2530–8600)	2330 (1220–6040)	6360 (3300–20000)	7440 (5130–12000)
**Mandenka**	6600 (2060–12300)	—	6930 (4320–*nd*)	4530 (2370–25200)	2750 (1400–13300)	1990 (582–25800)	12200 (8420–*nd*)
**San**	5340 (3440–12700)	3790 (1920–13800)	—	5820 (2900–13800)	7270 (4280–14500)	12700 (8260–31400)	5240 (3380–8820)
**Basque**	3250 (2050–14300)	2750 (2100–*nd*)	2490 (1620–7260)	—	2230 (1260–5090)	2120 (1110–12000)	2130 (1180–10900)
**Han Chinese**	2600 (1990–*nd*)	*nd* (7520–*nd*)	1880 (1230–3230)	1940 (1160–9300)	—	1050 (601–5710)	2490 (2390–6000)
**Melanesians**	2220 (106–4060)	293 (176–6000)	1330 (734–2710)	251 (133–2500)	858 (450–2300)	—	N/A
**PNG**	926 (425–2030)	342 (193–7710)	1090 (584–2000)	314 (149–962)	326 (180–1200)	N/A	—

Supplemental Table 2. Ancestral effective population sizes (N_A). Estimates of N_A are listed for each population pair. Central 95% confidence limits in parentheses.

	Biaka	Mandenka	San	Basque	Han Chinese	Melanesians	PNG
**Biaka**	—						
**Mandenka**	9980 (7150–13400)	—					
**San**	6620 (2130–14700)	9500 (7460–12400)	—				
**Basque**	11200 (8200–14900)	9970 (7780–13000)	10400 (7330–14300)	—			
**Han Chinese**	12800 (10100–16300)	11000 (8940–13900)	10200 (4490–18700)	11900 (8700–19900)	—		
**Melanesians**	12800 (8020–20900)	10600 (8150–17400)	11700 (6430–18600)	8830 (6390–14600)	12600 (7980–26400)	—	
**PNG**	10300 (6000–20100)	8220 (5390–16100)	9370 (5600–20700)	14500 (9060–28500)	9960 (6310–22400)	N/A	—

Supplemental Table 3. Proportion (S) of the ancestral deme founding each descendant population. Pairwise estimates of S are listed for each population on the horizontal. Central 95% confidence limits in parentheses.

	Biaka	Mandenka	San	Basque	Han Chinese	Melanesians	PNG
**Biaka**	—	0.999 (0.065–0.999)	0.001 (0.001–0.951)	0.049 (0.030–0.849)	0.008 (0.004–0.074)	0.632 (0.178–0.925)	0.002 (0.002–0.939)
**Mandenka**	0.001 (0.001–0.935)	—	0.901 (0.282–0.954)	0.021 (0.011–0.214)	0.006 (0.003–0.020)	0.002 (0.001–0.094)	0.006 (0.001–0.095)
**San**	0.999 (0.049–0.999)	0.099 (0.046–0.718)	—	0.154 (0.055–0.770)	0.656 (0.055–0.919)	0.963 (0.040–0.979)	0.791 (0.041–0.942)
**Basque**	0.951 (0.151–0.970)	0.979 (0.786–0.989)	0.846 (0.230–0.945)	—	0.003 (0.002–0.972)	0.999 (0.017–0.999)	0.006 (0.006–0.977)
**Han Chinese**	0.992 (0.926–0.996)	0.994 (0.980–0.997)	0.344 (0.081–0.945)	0.997 (0.028–0.998)	—	0.999 (0.040–0.999)	0.999 (0.909–0.999)
**Melanesians**	0.368 (0.075–0.822)	0.998 (0.906–0.999)	0.037 (0.021–0.960)	0.001 (0.001–0.983)	0.001 (0.001–0.960)	—	N/A
**PNG**	0.998 (0.061–0.998)	0.994 (0.905–0.999)	0.209 (0.022–0.959)	0.994 (0.023–0.994)	0.001 (0.001–0.091)	N/A	—

Supplemental Table 4. Pairwise interpopulation migration rates (m per generation, horizontal to vertical population).

Central 95% confidence limits in parentheses.

	Biaka	Mandenka	San	Basque	Han Chinese	Melanesians	PNG
**Biaka**	—	2.78×10^{-4} (1.29×10^{-4}–8.51×10^{-4})	8.59×10^{-5} (2.23×10^{-5}–4.42×10^{-4})	1.11×10^{-4} (3.67×10^{-5}–2.84×10^{-4})	3.04×10^{-5} (5.49×10^{-6}–4.76×10^{-4})	5.42×10^{-5} (9.60×10^{-6}–1.33×10^{-4})	4.20×10^{-5} (6.13×10^{-6}–1.40×10^{-4})
**Mandenka**	1.89×10^{-4} (5.26×10^{-5}–4.53×10^{-4})	—	7.15×10^{-5} (1.27×10^{-5}–2.85×10^{-4})	5.75×10^{-4} (2.23×10^{-4}–2.07×10^{-3})	2.21×10^{-4} (2.66×10^{-5}–1.77×10^{-3})	2.65×10^{-4} (5.20×10^{-5}–2.12×10^{-3})	1.52×10^{-4} (9.83×10^{-6}–8.44×10^{-4})
**San**	1.89×10^{-4} (1.76×10^{-5}–4.10×10^{-4})	2.02×10^{-6} (0–4.09×10^{-4})	—	4.15×10^{-5} (6.36×10^{-6}–1.28×10^{-4})	2.39×10^{-5} (5.44×10^{-6}–7.04×10^{-5})	1.16×10^{-7} (0–9.75×10^{-5})	5.78×10^{-8} (0–4.70×10^{-5})
**Basque**	4.28×10^{-5} (4.86×10^{-6}–1.50×10^{-4})	3.64×10^{-6} (1.91×10^{-6}–2.22×10^{-4})	1.16×10^{-7} (0–1.41×10^{-4})	—	9.17×10^{-4} (3.64×10^{-4}–2.15×10^{-3})	2.75×10^{-5} (4.34×10^{-6}–1.55×10^{-3})	2.52×10^{-5} (9.54×10^{-6}–1.41×10^{-3})
**Han Chinese**	7.52×10^{-6} (1.04×10^{-6}–7.76×10^{-5})	1.82×10^{-5} (3.30×10^{-6}–2.33×10^{-4})	4.63×10^{-5} (4.68×10^{-6}–1.38×10^{-4})	1.37×10^{-4} (1.56×10^{-5}–7.79×10^{-4})	—	3.47×10^{-7} (0–6.64×10^{-4})	3.49×10^{-5} (5.32×10^{-6}–2.73×10^{-4})
**Melanesians**	1.16×10^{-7} (0–9.17×10^{-5})	1.42×10^{-4} (4.05×10^{-5}–2.04×10^{-3})	1.85×10^{-4} (6.33×10^{-5}–5.01×10^{-4})	1.33×10^{-3} (1.76×10^{-4}–5.35×10^{-3})	5.56×10^{-4} (2.07×10^{-4}–1.99×10^{-3})	—	N/A
**PNG**	1.42×10^{-4} (5.87×10^{-7}–6.02×10^{-4})	4.66×10^{-4} (1.08×10^{-4}–2.06×10^{-3})	1.29×10^{-4} (6.97×10^{-5}–3.27×10^{-4})	1.09×10^{-3} (3.28×10^{-4}–3.29×10^{-3})	3.61×10^{-3} (1.14×10^{-3}–1.18×10^{-2})	N/A	—

Supplemental Table 5. Population split times (t, thousands of years). Central 95% confidence limits in parentheses.

Abbreviations: *nd*, not determined.

	Biaka	Mandenka	San	Basque	Han Chinese	Melanesians	PNG
**Biaka**	—						
**Mandenka**	48.7 (25.7-140)	—					
**San**	50.0 (33.6-*nd*)	46.8 (30.6-111)	—				
**Basque**	61.4 (36.9-230)	23.4 (13.5-78.4)	83.0 (51.4-339)	—			
**Han Chinese**	27.7 (17.7-73.9)	15.5 (9.13-29.3)	151 (87.7-300)	85.6 (45.2-228)	—		
**Melanesians**	103 (57.4-*nd*)	7.20 (2.71-68.4)	68.5 (39.1-209)	6.00 (4.33-*nd*)	79.0 (26.8-238)	—	
**PNG**	73.9 (42.1-300)	17.6 (8.52-80.0)	*nd*	119 (48.1-331)	43.4 (24.9-168)	N/A	—

Supplemental Table 6. Consistency between simulated distributions and observed summary statistics of the population site frequency spectrum across 20 loci. 95% confidence intervals apply a multiple-test Bonferroni correction to accommodate an experiment-wise type-I error rate, α, of 0.05.

	θ_W		θ_π		Tajima's D	
	Distribution*	Outliers†	Distribution	Outliers	Distribution	Outliers
Biaka	20	*0*	20	*0*	19	*1*
Mandenka	19	*1*	19	*1*	20	*0*
San	20	*0*	20	*0*	20	*0*
Basque	20	*0*	20	*0*	19	*1*
Han	20	*0*	18	*2*	19	*1*
Oceanians	20	*0*	20	*0*	20	*0*

* Number of loci within corrected central 95% confidence interval of the simulated distributions.
† Number of loci outside corrected central 95% confidence interval of the simulated distributions.
